# Impact of the Heat Treatment Duration on Color and Selected Mechanical and Chemical Properties of Scots Pine Wood

**DOI:** 10.3390/ma15155425

**Published:** 2022-08-06

**Authors:** Magdalena Piernik, Magdalena Woźniak, Grzegorz Pinkowski, Kinga Szentner, Izabela Ratajczak, Andrzej Krauss

**Affiliations:** 1 Department of Woodworking Machines and Fundamentals of Machine Design, Faculty of Forestry and Wood Technology, Poznań University of Life Sciences, Wojska Polskiego 38/42, 60-637 Poznań, Poland; 2 Department of Chemistry, Faculty of Forestry and Wood Technology, Poznań University of Life Sciences, Wojska Polskiego 75, 60-625 Poznań, Poland

**Keywords:** heat treatment, *Pinus sylvestris* L., color, mechanical properties, ATR-FTIR, chemical composition

## Abstract

The aim of this study was to assess the effect of the duration of heat treatment on changes in the color, as well as the chemical and mechanical properties of Scots pine sapwood. An important element of the research was to obtain the assumed temperature in the entire volume of samples. Quantitative changes in color and its components were recorded, while mechanical properties were determined in tests of compressive strength parallel and perpendicular to the grain, longitudinal tensile strength and modulus of elasticity and impact strength. The novelty of the research was to determine the above-mentioned parameters for twin samples with identical moisture contents. Chemical analyses were conducted on heat-treated wood that was subjected to heat treatment at 220 °C for a period from 1 to 8 h. Extension of the heat treatment duration resulted in the increasing darkening of the wood, as well as a further reduction in the impact strength and tensile strength parallel to the grain by approx. 40 and 50%, respectively, compared to the control wood, but also compared to heat-treated wood for a shorter treatment duration. The heat treatment of wood caused changes in the contents of the wood components, as well as the elemental composition in the heat-treated wood, compared to the control pine. The changes in the structure of the heat-treated wood were confirmed by the attenuated total reflectance Fourier transform infrared spectroscopy (ATR-FTIR). Observed quantitative changes in the main wood components, its structural changes, as well as wood decomposition and increased crystallinity of cellulose explain significant changes in both the mechanical properties and the color of heat-treated wood.

## 1. Introduction

In the course of heat treatment, significant qualitative and quantitative changes take place in the chemical composition of wood (e.g., a decrease in the contents of carbohydrate components of a low degree of polymerization; the depolymerization of cellulose and a reduction in its degree of crystallinity), which manifest, for example, in the loss of mass and changes in the color of the wood, while they also affect its prospective properties. The impact of heat treatment causes changes in the physico-mechanical properties of wood as a result of the degradation of one or more chemical cell wall components (cellulose, hemicelluloses, lignin) [1,2,3,4,5,6].

Changes in the physical properties of heat-treated wood are reflected in increased dimensional stability, reduced hygroscopicity, decreased absorbability and darkening of the wood color. Heat treatment leads to the improvement of the natural durability of wood, which is essential in the case of European species, considered to exhibit relatively low resistance to atmospheric conditions. These properties, as well as a dark color of heat-treated wood, resembling that of exotic species, indicate the potential applicability of heat-treated wood as their substitute [5,6,7,8,9,10].

Moreover, heat treatment has a considerable effect on the mechanical properties of wood, which are closely related to wood water content. Heat-treated wood contains less bound water compared to control wood. For this reason, an assessment of properties in the case of heat-treated wood needs to take into consideration the moisture content of the compared wood material. Wood that is subjected to heat treatment is generally characterized by reduced mechanical properties, increased brittleness and susceptibility to cracking when compared to control wood. An increase in treatment temperature above 150 °C results in a deterioration of longitudinal tensile strength and bending strength, as well as an increase in compressive strength parallel to the grain and hardness [11,12,13,14,15].

The natural color of wood also changes markedly as a result of heat treatment. The intensity of its changes depends among other things on changes in the chemical composition of wood, the temperature and the time of treatment. The higher the temperature and the longer the heat treatment duration, the darker the wood color, particularly in the early wood zone of softwood species. A visual effect of the heat treatment process is related to wood darkening, which is related to the process conditions. At a constant heat treatment duration an increase in treatment temperature determines the intensity of changes to a darker color [6,16,17,18,19]; however, a review of the literature provides ambiguous data whether and to what degree the extension of heat treatment duration at a constant temperature at the same time changes the color and properties of the wood.

The boundary temperature for the heat-treated wood process under commercial conditions (in industrial practice) is 220 °C. At this temperature, considerable changes occur in the structure, mechanical properties, as well as the natural color of wood. The immediate goal of the study was to determine how extension of the heat treatment duration affects changes in the color, physico-mechanical and chemical properties of Scots pine sapwood.

Results concerning changes in the color and mechanical parameters of wood that is subjected to heat treatment at a high temperature (as compared to that typically used in practice for heat-treated wood) may indicate an optimal treatment duration to ensure the expected change in color, while preserving possibly high values of the wood mechanical parameters. The obtained new data in this respect should facilitate the reduction in treatment duration at the assumed heat treatment temperature, held constant throughout the process. In this way, it should contribute to improved economic results of the wood heat treatment process.

## 2. Materials and Methods

### 2.1. Materials

The material was collected from the outer sections of a balk of *Pinus sylvestris* L. of 63 mm in thickness, cut above breast height diameter from the butt end of a log from a tree aged approx. 100 years. The average width of annual growth rings in this stem was 2.3 mm, while the share of late wood in the annual growth increments was 31.6%. The density of the Scots pine sapwood that was used for the analyses was 530 kg/m^3^.

Defect-free wood was used to obtain tangentially oriented slats, which were subsequently cut longitudinally into two parts to produce pairs of twin battens. Afterwards, they were planed to the transverse dimensions of 20 × 20 mm and then cut into 330 mm long sections. The sections were conditioned until a moisture content of approx. 7% was achieved, then half of them were subjected to heat treatment and the other half constituted the control. For these sections, samples were obtained with shapes and dimensions that were adequate to test the mechanical properties of wood.

### 2.2. Methods

#### 2.2.1. Heat Treatment of Wood

Heat-treated wood was run under laboratory conditions in the atmosphere of superheated steam, which was introduced to the heated chamber when the temperature inside the wood samples reached 130 °C (the ThermoWood^®^ procedure), in accordance with the methodology that was adopted in earlier such research. The adopted procedure ensured an identical temperature of 220 °C throughout the entire sample volume for a period of 1, 2, 4, 6 and 8 h, respectively [20,21].

#### 2.2.2. Physical and Mechanical Properties

The physical and mechanical properties were determined in samples of heat-treated wood and twin samples of control wood of comparable moisture content.

Wood color was determined according to PN-ISO 7724-3:2003 [22] using a DataColor TOOLS 600 spectrophotometer (Datacolor Company, Lawrenceville, NJ, USA), recording coordinates in the CIELab system. The measurements were taken on the radial surface of the sections, always at the same four points before and after heat treatment, and on twin sections. The results were expressed by the number of units of total color difference—∆E.

(1) $$\Delta E=\sqrt{\Delta L^{2}+\Delta a^{2}+\Delta b^{2}}$$

- ΔL—the difference in the lightness
- Δa; ∆b—the chromatic coordinates.

The moisture content was determined according to ISO 13061-1 (2014) [23]. The equilibrium moisture content of heat-treated wood at a temperature of 220 °C for 1, 2, 4, 6 and 8 h amounted on average to 3.6%, while that of the control samples was 3.9%.

Density was determined according to ISO 13061-2 (2014) [24].

The mechanical properties were tested following the requirements of respective standards using a ZWICK Z050TH universal strength testing machine equipped with a ZWICK 066550.02 extensometer (Zwick/Roell, Ulm, Germany). The machine software, apart from the graphical recording of the test process, also made it possible to calculate the values of strength and modulus of elasticity (MOE).

Longitudinal compressive strength (CS_L_) was determined according to ISO 13061-17:2017 [25], while compressive strength perpendicular to the grain (so-called stress at proportionality limit), in the tangential direction CS_T_ and the radial direction CS_R_ was tested according to ISO 13061-5:2020 [26].

Longitudinal tensile strength (TS_L_) was determined according to PN-D-04107:1981 [27] on double-sided shoulder samples of 16 (T) × 20 (R) × 160 (L) mm, narrowed in the middle part at a length of 40 mm to the tangential dimension of 2.5 mm. Additionally, MOE_L_ was also recorded.

The impact strength (IS) of wood was determined according to PN-D-04104:1979 [28] on rectangular samples of 10 (T) × 10 (R) × 110 (L) mm using a Louis Schopper pendulum impact machine (support spacing of 70 mm) (Werkstoffprufmaschine Leipzig GmbH, Leipzig, Germany).

#### 2.2.3. Chemical Analysis

The samples of control and heat-treated wood were ground in a Fritsch type 15 laboratory mill (IKA Werke, Staufen im Breisgau, Germany) to sawdust with a particle size below 0.50 mm and used for all chemical analyses.

The chemical composition of wood was assayed according to the TAPPI standards. The content of extractives in the wood samples was determined in accordance with TAPPI T 204 cm-07 [29]. The cellulose content in the wood samples was assayed according to Seifert [30], the lignin content by applying the TAPPI T 222 om-06 standard [31], and the holocellulose content was assayed based on TAPPI T 9 wd-75 [32]. The content of hemicelluloses was calculated from the difference between holocellulose and cellulose.

The elemental composition (contents of carbon, nitrogen, hydrogen and oxygen) of the wood samples was determined using a Flash 2000 elemental analyzer (Thermo Fisher Scientific, Waltham, MA, USA).

The attenuated total reflectance Fourier transform infrared spectroscopy (ATR–FTIR) was applied to analyze changes in the wood structure after heat treatment using a Nicolet iS5 spectrometer (Thermo Fisher Scientific, Waltham, MA, USA). The obtained spectra were used to calculate the total crystalline index (TCI, H_1372_/H_2885_) [33]; the lateral order index (LOI, A_1427_/A_896_) [34]; and the hydrogen bond intensity (HBI, A_3400_/A_1320_) [35].

#### 2.2.4. Statistical Analysis

Statistical analyses included a factorial one-way ANOVA, followed by Tukey’s honest significant difference (HSD) test at α = 0.05. All the statistical analyses were performed using the TIBCO Software Inc. Statistica version 13.1 (Palo Alto, CA, USA).

## 3. Results and discussion

### 3.1. Wood Color

**Figure 1:**
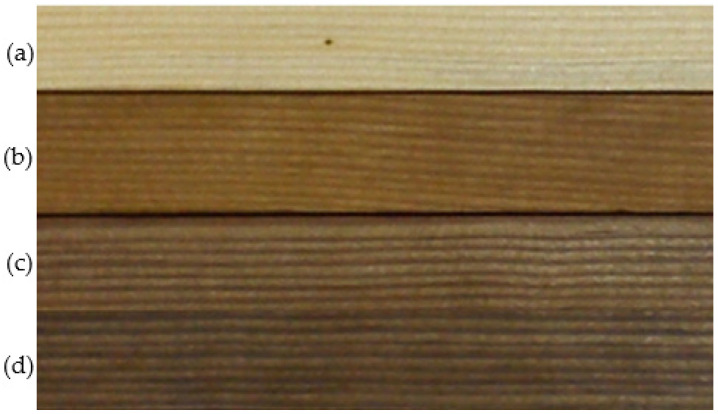
Sample surface—control (**a**) and heat-treated wood (constant temperature of heat treatment—220 °C) at a heat treatment duration of 1 h (**b**); 2 h (**c**); 8 h (**d**).

Heat treatment causes changes in wood color parameters and their intensity is affected by the extension of treatment time. The results are shown in Table 1 and Figure 1. The color of heat-treated wood darkens with extending the heat treatment duration.

Table 1 presents mean values and standard deviations (±σ_n−1_) of recorded parameters.

Extension of the heat treatment duration (within the range from 1 to 8 h) leads to an increase in ∆E by approx. 10 units, i.e., by 20%; a decrease in ∆L by approx. 14 units (35%); as well as a reduction in the values of coordinates ∆a and ∆b by approx. 2 and 10 units, i.e., by 17 and 41%, respectively. Color changes are important because ∆E is greater than 3 units, the value at which the variation of wood color is considered perceptible to the human eye [36]. The results are shown in Table 1. It was observed that with an extension of heat treatment time the wood color becomes darker (∆E), less red (∆a) and less yellow (∆b), while the lightness decreases, as shown by the difference in lightness (∆L). The decrease in lightness (∆L) resulting in total color difference (∆E) and accelerated darkening when heat treatment exceeded 200 °C was previously reported by González-Peña and Hale [19], and Bekhta and Niemz [13].

With an extension of heat treatment duration, an upward trend is observed for ∆E and a downward trend for ∆L, ∆a and ∆b. The level of the total change in wood color (∆E) it determined mainly by the parameter, whose value undergoes the greatest changes, i.e., ∆L. Changes in the total color difference (∆E) and its components in the function of heat treatment duration were approximated by linear functions. The results are shown in Table 2.

**Figure 2:**
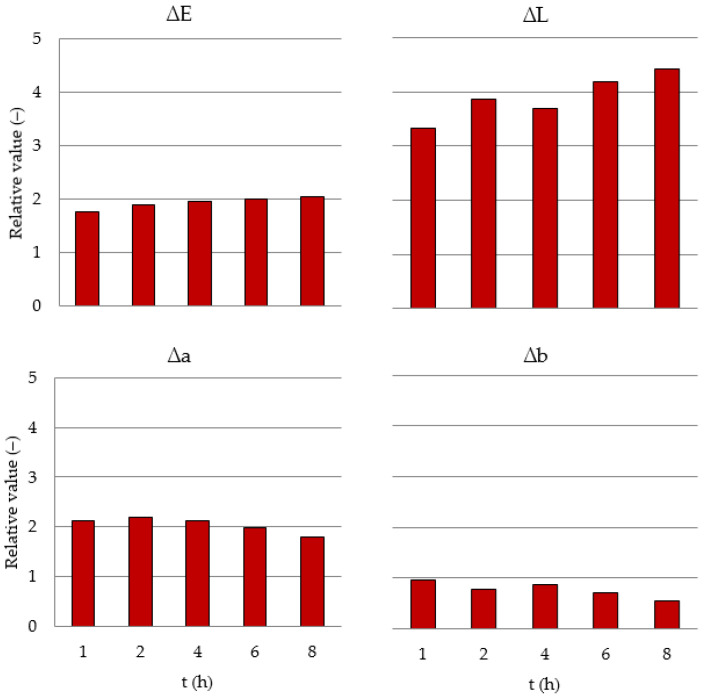
Relation to controlled value (in proportion) versus t (h).

Heat treatment causes an increase in the relative values of the investigated parameters of wood color (relative value = value of the investigated property in heat-treated wood related to the value of this property determined on twin samples of control wood). With an extension of heat treatment duration, the relative values of parameters ∆E and ∆L increase, while those of ∆a and ∆b decrease. After 8 h of heat treatment, the value of color parameter ∆E increases by 100%, ∆L by 340 % and ∆a by 90%, whereas the value of ∆b decreases by 50% in relation to the values of the respective parameter in wood that is not subjected to heat treatment. The results are shown in Figure 2.

### 3.2. Mechanical Properties

Heat treatment leads to a reduction in the longitudinal compressive strength of wood (CS_L_). The mean value of this strength in heat-treated wood is 62.5 MPa, while in control wood it is 70.2 MPa. The results are given in Table 3. The decrease in CS_L_ of heat-treated Scots pine wood was observed by Korkut et al. [37] and González-Peña and Hale [11].

Table 3 and the following presents mean values and standard deviations (±σ_n−1_) of recorded parameters.

**Figure 3:**
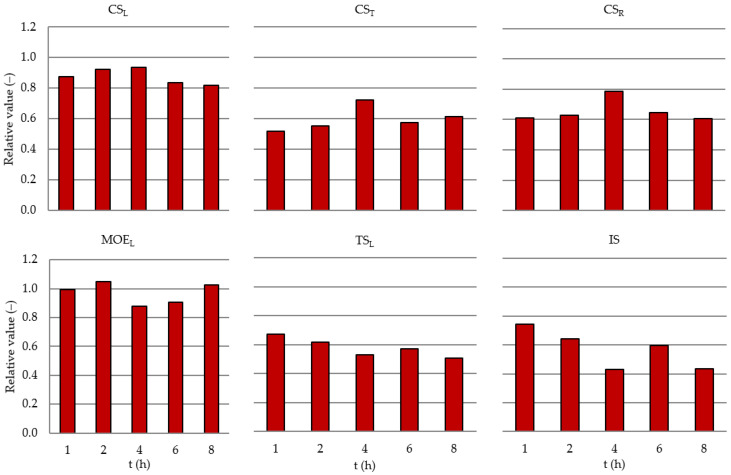
Relation to controlled value (in proportion) versus t (h). Each represents the mean value of *n* samples. IS, *n* = 20; CS_L_, *n* = 10; CS_T_, *n* = 10; CS_R_, *n* = 10; TS_L_, *n* = 10; MOE_L_, *n* = 10.

The analysis of changes in the relative compressive strength parallel to the grain in heat-treated wood indicates a slight effect of heat treatment duration. Heat-treated wood, compared to control wood, exhibits on average 12% lower longitudinal compressive strength, regardless of heat treatment duration. The results are given in Figure 3.

Heat treatment also causes a decrease in the compressive strength of wood perpendicular to the grain. The mean value of CS_T_ in heat-treated wood is 4.5 MPa, while that of control wood is 7.5 MPa. The mean value of CS_R_ for heat-treated wood is 2.4 MPa, whereas in control wood it is 3.8 MPa. However, no significant differences were found after an extension of the heat treatment duration. The results are shown in Table 3.

Compared to control wood, the average reduction in compressive strength in the tangential direction is 41%, while in the radial direction it is 35%. The results are given in Figure 3.

The analysis of changes in Young’s modulus along the grain indicates a lack of a marked effect of heat treatment duration on the modulus value (Table 3). The mean value for heat-treated wood is 14.3 GPa, compared to 13.9 GPa for control wood. A lack of any relationship between wood heat treatment duration and the values of the modulus of elasticity parallel to the grain is also confirmed by the analysis of relative values for this parameter (Figure 3).

The impact strength of heat-treated wood with an extension of heat treatment time decreases from 2.8 to 1.7 J/cm^2^. The mean value of impact strength for control wood is 3.9 J/cm^2^. Extension of the treatment time causes a decrease in the relative values of impact strength by 24% in the case of a 1-h heat treatment duration, and this is up to 58% for a heat treatment duration of 8 h (Figure 3). A decrease in the IS after the heat treatment corresponds with other research [11,19,37].

A reduction in strength at dynamic loading indicates an increase in the brittleness of heat-treated wood, while an extension of heat treatment time considerably intensifies the decrease in impact strength. This is confirmed by FTIR results, particularly the recorded increase in the degree of the crystallinity of cellulose. Changes in wood structure that take place at a temperature above 150 °C are significant in terms of mechanical properties [38]. A considerable reduction in the impact strength of heat-treated wood was also observed in earlier studies [11,12].

Tensile strength parallel to the grain in heat-treated wood decreases with an extension of the heat treatment time (Table 3). An analysis of relative values confirms this observation and indicates a considerable reduction in tensile strength parallel to the grain in the case of heat-treated wood at 220 °C, accounting for 68 to 50% strength of control wood (Figure 3). This may be explained by structural changes, decomposition and the increased brittleness of wood as a result of heat treatment.

The investigated mechanical properties varied little in the analyzed wood samples. The coefficient of variation (CV) of the control wood ranged from 4% (CS_T_) to 14% (IS), similar to heat-treated wood, where it was from 3% (CS_T_) to 25% (IS).

The testing results show that extension of the heat treatment duration over 2 h at 220 °C does not cause greater color intensity (further darkening) of Scots pine sapwood. An analysis of the effect of heat treatment duration on the mechanical properties of wood shows that reduced compressive strength parallel and perpendicular to grain is more or less identical and does not depend on heat treatment duration within the range of 1 up to 8 h. Moreover, the applied heat treatment has practically no effect on changes in the modulus of elasticity. In contrast, impact strength and tensile strength parallel to grain starting from the 4th h of the heat treatment process are considerably reduced on average by half, compared to the control wood. These results indicate that in the heat treatment of pine sapwood at the temperature of 220 °C to obtain intensive darkening at a relatively small deterioration of impact strength and tensile strength parallel to grain, the treatment duration may be reduced to approx. 2 h.

### 3.3. Chemical Composition of Heat-Treated Wood

The heat treatment of pine wood caused changes in the contents of the main wood components, as presented in Table 4.

Extending the heat treatment duration did not significantly affect the content of the extractives soluble in ethanol, used as a solvent. The content of extractives decreased by about 18% in heat-treated wood compared to the control wood and was similar in all variants of heat treatment duration (Table 4). According to the statistical analysis, wood composition after 6 h of heat treatment was characterized by a lower content of extractives, compared to heat-treated wood in other time variants. The decrease in the extractive contents resulted from the removal of volatile substances, lignin degradation products and carbohydrates in the extraction mixture.

During heat treatment, the content of cellulose in heat-treated wood increased from 45.24% to 57.28% (Table 4). The apparent relative increase in cellulose content can be attributed to structural changes and the cross-linking reaction of the polymer.

Among all the analyzed wood components, the low molecular weight carbohydrates are the most susceptible to heat treatment. The progress of the degradation depends on the heat treatment duration.

The decrease in hemicelluloses contents in heat-treated wood for 4 h was about 85% of the content in control wood (Table 4).

Lignin systems were characterized by greater thermal stability compared to the carbohydrates. The lignin content in heat-treated wood in the period from 1 to 8 h showed a relative increase (about 16.9%) compared to the control wood. Moreover, as shown by the statistical analysis, the lignin content in heat-treated wood is related to the duration of the heat treatment. The increase in lignin content could be connected with the condensation reaction [39,40], while condensation is the effect of breaking the β-O-4 bonds in lignin and the methoxy groups. The present results of lignin contents at various thermal processing times correspond to the obtained results of wood color changes (Table 1). The changes in the structural components of wood after heat treatment correspond with those that are presented in other studies [41,42].

The ratio of cellulose to lignin (C/L) and of holocellulose to lignin (H/L) in heat-treated wood are indicators of wood decomposition after heat treatment.

The values of the C/L and H/L ratios in heat-treated and control wood are presented in Table 4. The H/L ratio ranged from 3.25 (for the control sample) to 1.89 (for wood after 8 h heat treatment). A significant reduction in the value of this index is confirmed by the increase in the degradation progress of low molecular weight carbohydrate components in wood during heat treatment.

A slight decrease in the ratio of cellulose to lignin systems (C/L) ranging from 1.68 (control wood) to 1.57 (wood after 8 h treatment) during heat treatment indicates a greater thermal stability of cellulose compared to hemicelluloses. The slight reduction in the C/L ratio is due to the disturbance of the amorphous regions of cellulose during heat treatment.

### 3.4. Ultimate Analysis of Heat-Treated Wood

The ultimate analysis (nitrogen, carbon, hydrogen and oxygen contents) of the control and heat-treated wood is presented in Table 5.

The nitrogen and hydrogen concentrations were similar in the control and heat-treated wood, regardless of the heat treatment duration. Heat treatment caused an increase in carbon concentration and a decrease in oxygen concentration when compared to control wood, which was confirmed by the statistical analysis. Moreover, the changes in the C and O contents were especially evident in the case of heat-treated wood for 6 and 8 h. The increase in carbon content and decrease in oxygen concentration in heat-treated wood was also observed in the case of heat-treated oak, poplar, beech, ash, pine or fir wood [43,44,45]. The reduction in O content in heat-treated wood could be connected with decarboxylation (cleavage of acetic acid from hemicelluloses) [44]. Heat treatment caused a reduction in O/C and H/C ratios compared to control wood, caused mainly by the degradation, decarboxylation and deacetylation of polysaccharide [46]. The decrease in the O/C and H/C ratios of heat-treated wood was previously reported in the available literature based on X-ray photoelectron spectroscopy analysis and elemental analysis (contents of C, H, N and O) [43,47].

### 3.5. Infrared Spectroscopy of Heat-Treated Wood

**Figure 4:**
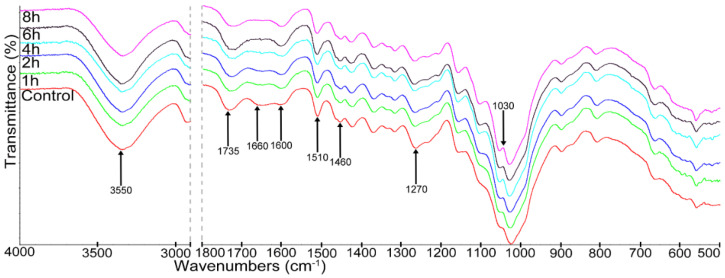
ATR-FTIR spectra of control and heat-treated wood.

Changes in the structure of wood caused by heat treatment carried out at different times were determined using the ATR-FTIR analysis, the results of which are presented in Figure 4 in the form of spectra.

The heat treatment of pine wood for a period of 1–8 h caused a reduction in the intensity of the band at 3550 cm^−1^ ascribed to hydroxyl groups, compared to control wood [48]. In the spectra of heat-treated wood, the reduction in the intensity bands was observed at 1735 cm^−1^ (C=O ester non-conjugated carbonyl group) and at 1655 cm^−1^ (C-O in quinones coupled with C=O stretching in various groups), related to hemicelluloses degradation [42]. The decrease in the intensity of lignin characteristic bands at 1510 cm^−1^ (C=C stretching of the aromatic skeleton); 1460 cm^−1^ (deformation of the C-H bond of xylan); 1270 cm^−1^ (guaiacyl ring breathing); and 1030 cm^−1^ (C-O ester stretching vibrations in methoxyl and β-O-4 linkages) was also visible in the spectra of heat-treated wood samples, compared to the spectra of the control wood [42,49,50]. These changes observed in the spectra of heat-treated wood samples in comparison with the spectrum of control wood were connected with lignin degradation during heating. In turn, the increase in band intensity at 1600 cm^−1^ in wood after heat treatment was related to the increase in aromatic rings in the wood components after their degradation [50]. The FTIR results showed that the heat treatment of pine wood caused changes in the structure of wood compared to that in the control sample. On the other hand, extending the time of heat-treated wood had a slight effect on changes taking place in the structure of heat-treated wood.

The applied FTIR analysis is a very simple and useful method of assessing structural changes in wood. The total crystallinity index (TCI), the lateral order index (LOI) and the hydrogen bond intensity (HBI) were determined and are presented in Table 6.

The LOI index correlated with the overall degree of cellulose order in wood and ranged from 0.62 to 0.68. In turn, the TCI index changed from 1.3 (for control wood) to 1.5 (for wood after 8 h heat treatment), which indicated an increase in the crystallinity of cellulose in wood. However, no significant differences in this parameter were found when extending the time of wood heat treatment. The change in crystallinity may be determined by the reorientation or rearrangement of quasicrystalline amorphous areas of cellulose or may be caused by crystallization in hemicelluloses [51,52]. The TCI index that is used to assess changes in the crystallinity of samples is a very useful tool because it correlates well with the results of X-ray analysis [53]. The increase in crystallinity is a very important parameter, as it indicates greater thermal stability and greater strength of the analyzed materials [54]. This is explained by changes in the strength of wood after heat treatment in our study.

The hydrogen bond intensity (HBI) ranged from 1.34 to 1.05. This change is related to the crystal system and the degree of intermolecular regularity [35].

## 4. Conclusions

This paper presents the results of analyses concerning the physico-mechanical and chemical properties of the Scots pine (*Pinus sylvestris* L.) sapwood, which was subjected to heat treatment at the temperature of 220 °C with a duration of 1, 2, 4, 6 and 8 h. An important element of this study was to run the heat treatment process so as to ensure the temperature of 220 °C over the entire sample volume and to determine the wood properties (color, modulus of elasticity, tensile strength, compressive strength and impact strength) on twin samples of identical moisture content. It was crucial to gain information on how multiple variants of heat treatment duration intensify changes in wood properties. The color of heat-treated wood at 220 °C compared to that of control wood undergoes considerable changes (darkening), and after the first hour of treatment, ∆E increases by 72%, while after the second hour it increases by 93%.

After 1 h of heat treatment a considerable color change was observed to over a three-fold decrease in lightness, as well as a deterioration of the mechanical properties in wood: longitudinal tensile strength by 33%; compressive strength in the tangential direction by 45%; compressive strength in the radial direction by 39%; compressive strength parallel to the grain by 15%; as well as impact strength by 25%, in comparison to the twin samples of control wood. In contrast, no effect of heat treatment was observed on the modulus of elasticity. Extension of the treatment duration from 1 to 8 h causes a relatively limited darkening of wood and parameter ∆E increases within this range by 20%. Changes in color parameters ∆E, ∆L, ∆a and ∆b in the function of heat treatment duration may be well approximated using linear functions.

With an extension of heat treatment duration, a further marked reduction was observed in impact strength and longitudinal tensile strength, while no effect of extended heat treatment duration was found on the compressive strength or modulus of elasticity in heat-treated wood. Analyses confirmed the opinion on a deterioration of strength and an increase in the brittleness of heat-treated wood. In contrast, these analyses contradicted reports that heat treatment enhances compressive strength parallel to the grain [12,55].

The heat-treated of pine sapwood at 220 °C shows lower contents of extractives, holocellulose and hemicelluloses, as well as greater contents of cellulose and lignin compared to control wood. With an extension of the heat treatment duration in heat-treated wood, the contents of cellulose and lignin increase, levels of hemicelluloses and holocellulose decrease, while contents of extractives do not depend on heat treatment duration. Heat-treated wood is characterized by increased cellulose crystallinity, regardless of heat treatment duration.

## Figures and Tables

**Table 1:** Parameters of color change in heat-treated wood at different treatment time and at constant temperature.

Time t (h)	Parameters of Color (–)			
	∆L	∆a	∆b	∆E
1	−41.49 ± 2.72	10.10 ± 0.49	23.27 ± 1.15	48.40 ± 2.09
2	−49.53 ± 2.01	10.52 ± 0.51	19.18 ± 1.02	54.17 ± 1.68
4	−47.93 ± 1.21	10.19 ± 0.49	20.18 ± 1.02	53.01 ± 1.00
6	−52.19 ± 1.54	9.45 ± 0.51	17.24 ± 1.11	55.79 ± 1.20
8	−55.85 ± 1.31	8.41 ± 0.36	13.73 ± 1.02	58.15 ± 1.02
Control *	−12.60 ± 0.73	4.75 ± 0.37	24.69 ± 0.95	28.13 ± 1.14

* Values of parameters of control wood are mean values from measurements of color in all control (twin) samples.

**Table 2:** Constants in the regression equation y = ax + b relating parameters of color to time of heat treatment.

Parameters of Color	Constants		Correlation Coefficient
	a	b	R
∆E	1.048	49.712	0.77
∆L	−1.574	−43.085	0.81
∆a	−0.267	10.872	0.76
∆b	−0.994	22.553	0.83

**Table 3:** Mechanical properties of wood.

Mechanical Properties	Heat Treatment Duration (h)					
	1	2	4	6	8	Control *
CS_L_ [MPa]	64.0 ± 10.5	64.9 ± 4.4	67.9 ± 3.4	59.9 ± 4.8	55.7 ± 2.3	70.2 ± 4.3
CS_T_ [MPa]	3.9 ± 0.5	4.2 ± 0.4	5.4 ± 0.5	4.3 ± 0.1	4.6 ± 0.2	7.5 ± 0.3
CS_R_ [MPa]	2.3 ± 0.3	2.4 ± 0.3	2.7 ± 0.3	2.5 ± 0.3	2.3 ± 0.2	3.8 ± 0.5
TS_L_ [MPa]	63.4 ± 8.0	58.5 ± 8.2	58.4 ± 11.9	55.0 ± 6.9	56.6 ± 15.6	100.1 ± 14.3
MOE_L_ [GPa]	12.6 ± 1.0	14.9 ± 0.7	16.2 ± 0.7	12.5 ± 0.9	15.4 ± 0.5	13.9 ± 1.4
IS [J/cm^2^]	2.81 ± 0.30	2.61 ± 0.37	1.54 ± 0.27	2.40 ± 0.34	1.72 ± 0.43	3.85 ± 0.55

* mean values of a given property were determined in all control wood samples (control).

**Table 4:** Contents of extractives and main components in heat-treated wood.

Content (%)	Heat Treatment Duration (h)					
	1	2	4	6	8	Control
Extractives	11.80 ^b^ ± 0.01	11.60 ^b,c^ ± 0.00	11.24 ^c^ ± 0.01	10.80 ^d^ ± 0.10	11.50 ^b,c^ ± 0.10	13.29 ^a^ ± 0.11
Cellulose	46.65 ^e^ ± 0.38	47.67 ^d^ ± 0.18	57.29 ^a^ ± 0.16	52.06 ^b^ ± 0.32	50.42 ^c^ ± 0.03	45.24 ^f^ ± 0.30
Hemicelluloses *	20.45	19.58	6.32	8.92	10.08	42.26
Holocellulose	67.10 ^b^ ± 0.11	67.26 ^b^ ± 0.10	63.61 ^c^ ± 0.09	60.98 ^d^ ± 0.06	60.50 ^d^ ± 0.33	87.48 ^a^ ± 0.08
Lignin	29.57 ^d^ ± 0.18	30.87 ^c^ ± 0.17	32.40 ^a,b^ ± 0.02	32.55 ^a^ ± 0.09	32.00 ^b^ ± 0.04	26.91 ^e^ ± 0.27
H/L	2.26	2.17	1.96	1.87	1.89	3.25
C/L	1.57	1.54	1.76	1.59	1.57	1.68

* Content calculated from the difference between holocellulose and cellulose. Values denoted with identical letters do not differ significantly.

**Table 5:** The ultimate analysis of control and heat-treated wood.

Content of Element (%)	Heat Treatment Duration (h)					
	1	2	4	6	8	Control
Nitrogen	0.057 ^b^ ± 0.001	0.058 ^a,b^ ± 0.003	0.061 ^a,b^ ± 0.001	0.060 ^a,b^ ± 0.000	0.067 ^a^ ± 0.001	0.062 ^a,b^ ± 0.003
Carbon	50.076 ^b^ ± 0.169	50.137 ^b^ ± 0.068	50.327 ^b^ ± 0.059	51.238 ^a^ ± 0.146	51.561 ^a^ ± 0.069	47.757 ^c^ ± 0.013
Hydrogen	6.075 ^a^ ± 0.022	6.124 ^a^ ± 0.003	6.111 ^a^ ± 0.008	6.037 ^a^ ± 0.001	6.028 ^a^ ± 0.009	6.170 ^a^ ± 0.069
Oxygen	43.382 ^b^ ± 0.134	43.431 ^c^ ± 0.087	43.261 ^d^ ± 0.016	42.414 ^e^ ± 0.143	42.124 ^f^ ± 0.046	45.831 ^a^ ± 0.080
O/C ratio	0.870	0.866	0.860	0.828	0.817	0.960
H/C ratio	0.121	0.122	0.121	0.118	0.117	0.129

Values denoted with identical letters do not differ significantly.

**Table 6:** Infrared crystallinity ratio (LOI and TCI) and hydrogen bond intensity (HBI) of cellulose in heat-treated wood.

	Heat Treatment Duration (h)					
	1	2	4	6	8	Control
TCI (1372/2885) *	1.43 ± 0.01	1.48 ± 0.02	1.46 ± 0.06	1.49 ± 0.05	1.50 ± 0.03	1.38 ± 0.06
LOI (1427/896) *	0.60 ± 0.02	0.65 ± 0.01	0.65 ± 0.09	0.68 ± 0.02	0.68 ± 0.05	0.62 ± 0.01
HBI (3400/1320)	1.25 ± 0.01	1.22 ± 0.03	1.18 ± 0.02	1.15 ± 0.01	1.05 ± 0.01	1.34 ± 0.03

* IR crystallinity ratio.

## Data Availability

The data presented in this study are available on request from the corresponding author.

## References

[1] Pétrissans M., Géradin P., El-Bakali I., Seraj M. (2003). Wettability of heat-treated wood. Holzforschung.

[2] Hill C.A.S. (2006). Thermal Modification of Wood. Wood Modification: Chemical, Thermal and Other Processes.

[3] Kollmann F., Fengel D. (1965). Changes in the chemical composition of wood by heat treatment. Holz Roh-Und Werkst..

[4] Hirai N., Sobue N., Asano I. (1972). Studies on piezoelectric effect of wood. IV. Effects of heat treatment on cellulose crystallities and piezoelectric effect of wood. Mokuzai Gakkaishi.

[5] Kymäläinen M., Mlouka S.B., Belt T., Merk V., Liljeström V., Hänninen T., Uimonen T., Kostiainen M., Rautkari L. (2018). Chemical, water vapour sorption and ultrastructural analysis of Scots pine wood thermally modified in high-pressure reactor under saturated steam. J. Mater. Sci..

[6] Sivrikaya H., Tesařová D., Jeřábková E., Can A. (2019). Color change and emission of volatile organic compounds from Scots pine exposed to heat and vacuum-heat treatment. J. Build. Eng..

[7] Altgen M., Hofmann T., Militz H. (2016). Wood moisture content during the thermal modification process affects the improvement in hygroscopicity of Scots pine sapwood. Wood Sci. Technol..

[8] Hillis W.E. (1984). High temperature and chemical effects on wood stability. Part 1: General considerations. J. Wood Sci. Technol..

[9] Ewert M., Scheiding W. (2005). Thermoholz in der Anwendung-Eigenschaften und Möglichkeiten. Holztechnologie.

[10] Pfriem A., Horbens M., Beyer M., Peters J. (2009). Untersuchung von Extraktstoffen aus termisch modifizierter Rotbuche (*Fagus sylvatica* L.) auf ihre fungizide Wirkung. Holztechnologie.

[11] González-Peña M.M., Hale M.D.C. The relationship between mechanical performance and chemical changes in thermally modified wood. Proceedings of the Third European Conference on Wood Modification.

[12] Boonstra M.J. (2008). A Two-Stage Thermal Modification of Wood. Ph.D. Thesis.

[13] Bekhta P., Niemz P. (2003). Effect of high temperature on the change in color, dimensional stability and mechanical properties of spruce wood. Holzforschung.

[14] Esteves B., Pereira H. (2009). Wood modification by heat treatment: A review. BioResources.

[15] Boonstra M.J., Van Acker J., Tjeerdsma B.F., Kegel E.F. (2007). Strength properties of thermally modified softwoods and its relation to polymeric structural wood constituents. Ann. For. Sci..

[16] Bourgois P.J., Janin G., Guyonnet R. (1991). The colour measurement–a fast method to study and to optimize the chemical transformations undergone in thermally-treated wood. Holzforschung.

[17] Brischke C., Walzbacher C.R., Brandt K., Rapp A.O. (2007). Quality control of thermal modified timber: Interrelationship between heat treatment intensities and CIE L*a*b* colour data on homogenized wood samples. Holzforschung.

[18] Johansson D., Morén T. (2006). The potential of colour measurement for strength prediction of thermally treated wood. Holz Roh-Und Werkst..

[19] González-Peña M.M., Hale M.D.C. (2009). Colour in thermally modified wood of beech, Norway spruce and Scots pine. Part 1: Colour evolution and colour changes. Holzforschung.

[20] Krauss A., Piernik M., Pinkowski G. (2016). Cutting power during milling of thermally modified pine wood. Drv. Ind..

[21] Piernik M., Rogoziński T., Krauss A., Pinkowski G. (2019). The influence of the thermal modification of pine (*Pinus sylvestris* L.) wood on the creation of fine dust particles in plane milling. J. Occup. Health.

[22] (2003). Farby i lakiery–Kolorymetria–Część 3: Obliczanie różnic barwy.

[23] (2014). Physical and Mechanical Properties of Wood—Test. Methods for Small Clear Wood Specimens—Part 1: Determination of Moisture Content for Physical and Mechanical Tests.

[24] (2014). Physical and Mechanical Properties of Wood—Test. Methods for Small Clear Wood Specimens—Part 2: Determination of Density for Physical and Mechanical Tests.

[25] (2017). Physical and Mechanical Properties of Wood—Test. Methods for Small Clear Wood Specimens—Part 17: Determination of Ultimate Stress in Compression Parallel to Grain.

[26] (2020). Physical and Mechanical Properties of Wood—Test. Methods For Small Clear Wood Specimens—Part 5: Determination of Strength in Compression Perpendicular to Grain.

[27] (1981). Drewno. Oznaczanie wytrzymałości na rozciąganie wzdłuż włókien.

[28] (1979). Drewno. Oznaczanie udarności i wytrzymałości na zginanie dynamiczne.

[29] (2007). Solvent Extractives of Wood and Pulp.

[30] Seifert K. (1960). Zur Frage der Cellulose-Schnellbestimmung nach der Acety-laceton-Methode. Papier.

[31] (2006). Acid-Insoluble Lignin in Wood and Pulp.

[32] (2006). Holocellulose in Wood.

[33] Nelson M.L., O’Connor R.T. (1964). Relation of certain infrared bands to cellulose crystallinity and crystal lat-tice type. Part I. Spectra types I, II, III and amorphous cellulose. J. Appl. Polym. Sci..

[34] Carrillo F., Colom X., Suñol J.J., Saurina J. (2004). Structural FTIR analysis and thermal characterisation of lyocell and viscose-type fibres. Eur. Polym. J..

[35] Oh S.Y., Yoo D.I., Shin Y., Seo G. (2005). FTIR analysis of cellulose treated with sodium hydroxide and carbon dioxide. Carbohydr. Res..

[36] Müller U., Rätzsch M., Schwanninger M., Steiner M., Zöbl H. (2003). Yellowing and IR-changes of spruce wood as result of UV-irradiation. J. Photochem. Photobiol. Biol..

[37] Korkut S., Akgül M., Dündar T. (2008). The effects of heat treatment on some technological properties of Scots pine (*Pinus sylvestris* L.) wood. Bioresour. Technol..

[38] Parysek M. (2010). Badania Zmian Składu Chemicznego Drewna Zmodyfikowanego Termicznie. Ph.D. Thesis.

[39] Hill C., Altgen M., Rautkari L. (2021). Thermal modification of wood—a review: Chemical changes and hygroscopicity. J. Mater. Sci..

[40] Rousset P., Lapierre C., Pollet B., Quirino W., Perre P. (2009). Effect of severe thermal treatment on spruce and beech wood lignins. Ann. For. Sci..

[41] Vybohova E., Balazova Z. (2018). The effect of heat treatment on the chemical composition of Ash wood. BioResources.

[42] Sikora A., Kačík F., Gaff M., Vondrová V., Bubeníková T., Kubovský I. (2018). Impact of thermal modification on color and chemical changes of spruce and oak wood. J. Wood Sci..

[43] Chaouch M., Dumarçay S., Pétrissans A., Pétrissans M., Gérardin P. (2013). Effect of heat treatment intensity on some conferred properties of different European softwood and hardwood species. Wood Sci. Technol..

[44] Boonstra M.J., Tjeerdsma B. (2006). Chemical analysis of heat treated softwoods. Holz Als Roh-Und Werkst..

[45] Mohareb A., Sirmah P., Pétrissans M., Gérardin P. (2012). Effect of heat treatment intensity on wood chemical composition and decay durability of *Pinus patula*. Eur. J. Wood Wood Prod..

[46] Willems W., Lykidis C., Altgen M., Clauder L. (2015). Quality control methods for thermally modified wood. Holzforschung.

[47] Wang W., Zhu Y., Cao J., Sun W. (2015). Correlation between dynamic wetting behavior and chemical components of thermally modified wood. Appl. Surf. Sci..

[48] González-Peña M.M., Curling S.F., Hale M.D.C. (2009). On the effect of heat on the chemical composition and dimensions of thermally-modified wood. Polym. Degrad. Stab..

[49] Miklečić J., Jirouš-Rajković V., Antonović A., Španić N. (2011). Discolouration of thermally modified wood during simulated indoor sunlight exposure. BioResources.

[50] Shen H., Cao J., Sun W., Peng Y. (2016). Influence of post-extraction on photostability of thermally modified Scots pine wood during artificial weathering. BioResources.

[51] Bhuiyan M.T.R., Hirai N., Sobue N. (2000). Changes of crystallinity of wood cellulose by heat treatment under dried and moist conditions. J. Wood Sci..

[52] Akgül M., Gümüskaya E., Korkut S. (2007). Crystalline structure of heat-treated Scot pine [*Pinus sylvestris* L.] and Uludag fir [*Abies nordmannina* (Stev.) subbsp. *bornmulleriana* (Mattf.)] wood. Wood Sci. Technol..

[53] Kasprzyk H., Wichlacz K. (2004). Some aspects of estimation of the crystallinity of gamma radiation wood cellulose by FTIR spectroscopy and X-ray diffraction techniques. Acta Sci. Pol. Silvarum.

[54] Poletto M., Zattera A.J., Forte M.M.C., Santana R.M.C. (2012). Thermal decomposition of wood: Influence of wood components and cellulose crystallite size. Bioresour. Technol..

[55] Grześkiewicz M., Dąbrowski P. (2004). Badanie wybranych właściwości fizycznych i mechanicznych drewna modyfikowanego-ThermoWood. Przemysł Drzewny.

